# Atypical Hemolytic Uremic Syndrome Triggered by COVID‐19 Infection in a Pediatric Patient With CD46 Mutation

**DOI:** 10.1002/ccr3.70308

**Published:** 2025-03-14

**Authors:** Parsa Lorestani, Parisa Maleki Dana, Mohamad Reza Tohidi

**Affiliations:** ^1^ Students Research Committee Kermanshah University of Medical Sciences Kermanshah Iran; ^2^ Research Center for Biochemistry and Nutrition in Metabolic Diseases Kashan University of Medical Sciences Kashan Iran; ^3^ Department of Pediatric Nephrology, Urology and Nephrology Research Center Kermanshah University of Medical Sciences Kermanshah Iran

**Keywords:** atypical hemolytic uremic syndrome, CD46 mutation, COVID‐19, eculizumab, thrombotic microangiopathy

## Abstract

Atypical hemolytic uremic syndrome (aHUS) is a rare form of thrombotic microangiopathy (TMA) that is considered life‐threatening and caused by dysregulation of the complement system. Here, we report a previously healthy 8‐year‐old boy who presented with features of aHUS 1 week after viral symptoms during the COVID‐19 pandemic. The patient was initially admitted for viral symptoms, pallor, edema, and urine color changes. Laboratory tests revealed anemia, thrombocytopenia, and elevated levels of creatinine and blood urea nitrogen (BUN). Despite fluid and electrolyte management, he developed pulmonary edema, necessitating hemodialysis and plasmapheresis. Genetic testing identified a homozygous pathogenic mutation in the CD46 gene, which encodes membrane cofactor protein (MCP). While initially responding to treatment, the patient experienced a relapse, requiring further interventions including eculizumab therapy. This case highlights the potential of COVID‐19 to trigger complement‐mediated TMA and emphasizes the importance of prompt diagnosis, genetic assessment, and targeted complement inhibition in managing aHUS.

**Summary**

- Atypical hemolytic uremic syndrome can be triggered by COVID‐19 in genetically predisposed individuals.
- Prompt recognition, comprehensive genetic testing, and targeted complement inhibition are crucial for managing this life‐threatening condition, especially in patients with CD46 mutations.

## 1 Introduction

Thrombotic microangiopathy (TMA) is a life‐threatening syndrome characterized by systemic microvascular occlusions. Atypical hemolytic uremic syndrome (aHUS) is a rare form of TMA primarily affecting the kidneys. The global prevalence of aHUS is not precisely known due to its rarity. Without treatment, up to two‐thirds of patients develop end‐stage renal disease following disease onset or relapse [1, 2]. Approximately 80% of cases present with the triad of thrombocytopenia, renal failure, and microangiopathic hemolytic anemia, with about 60% showing genetic predisposition [1].

The alternative pathway of the complement system is considered the primary cause of aHUS, with mutations in genes encoding membrane cofactor protein (CD46, MCP) and complement factor H (CFH) being the most common [1]. Recent studies have suggested that viral infections, including COVID‐19, may trigger aHUS in genetically susceptible individuals [3, 4].

Here, we describe a case of an 8‐year‐old male who presented with aHUS following COVID‐19 infection, with genetic testing revealing a homozygous pathogenic variant in the CD46 gene.

## 2 Case History/Examination

A previously healthy 8‐year‐old boy presented to our hospital with a 1‐week history of fever, cough, and fatigue consistent with a viral infection during the COVID‐19 pandemic. At the time of admission, he also showed pallor, periorbital edema, and cola‐colored urine.

## 3 Methods (Differential Diagnosis, Investigations, and Treatment)

### 3.1 Initial Investigations

Preliminary laboratory tests revealed:

- Hemoglobin: 7 g/dL
- Platelets: 80,000/μL
- Blood urea nitrogen: 60 mg/dL
- Creatinine: 2.5 mg/dL
- Lactate dehydrogenase: 1200 U/L
- Urinalysis: Positive for hematuria (50–100 red blood cells/high power field) and 2+ proteinuria
- Coomb's test: Negative
- C3 and G6PD levels: Normal

### 3.2 Differential Diagnosis

Based on the clinical presentation of microangiopathic hemolytic anemia, thrombocytopenia, and acute kidney injury, a presumptive diagnosis of aHUS was made. Other considerations included typical HUS, thrombotic thrombocytopenic purpura (TTP), and other forms of TMA.

### 3.3 Initial Treatment

1. During the first 24 h, despite normal blood pressure, the patient was oliguric (urine output less than 1 cc/kg/h).
2. On the second day, the patient's clinical status deteriorated as evidenced by worsening oliguria and the development of pulmonary edema due to fluid overload. Emergent hemodialysis became necessary.
3. The patient underwent five sessions of plasmapheresis over the course of 1 week.

### 3.4 Further Investigations

1. Complement regulatory protein testing: Normal levels of Factor H, Factor I, and ADAMTS13; an abnormal CD46 (MCP) level detected.
2. Genetic testing: Whole exome sequencing was performed using the Illumina platform with Agilent SureSelect V7 library preparation kit at 150X depth. This analysis confirmed a homozygous Likely Pathogenic variant in the CD46 gene: NM_172351.3:c.565T>G (p.Tyr189Asp). This variant is associated with “Hemolytic uremic syndrome, atypical, susceptibility to, 2 (AR)” and has been reported in the ClinVar database.
3. Flow cytometry: Total CD46 expression at 45.0% (normal range 64%–99%), further supports the functional impact of the homozygous CD46 mutation.

The complete genetic findings, including both major and secondary variants identified in this patient, are summarized in Table 1. These results were crucial in confirming the diagnosis of aHUS and guiding subsequent treatment decisions.

**TABLE 1:** Genetic findings in a pediatric patient with aHUS triggered by COVID‐19 infection.

Genetic findings		
Major finding	Gene	CD46
	cDNA	NM_172351.3 c.565T>G
	Protein	p.Tyr189Asp
	Zygosity	Homozygous
	Significance	Likely Pathogenic
	Phenotype	Hemolytic uremic syndrome, atypical, susceptibility to, 2 (AR)
Secondary finding	Gene	DNAH9
	cDNA	NM_001372_4 c.4995_4998del
	Protein	p.Phe1666ValfsTer10
	Zygosity	Heterozygous
	Significance	Likely Pathogenic
	Phenotype	Ciliary dyskinesia, primary, 40 (AR)

*Note:* The table shows major and secondary genetic variants identified, including a pathogenic variant in the CD46 gene associated with aHUS.

## 4 Results (Outcome and Follow‐Up)

Following plasmapheresis, the patient's hemoglobin level stabilized, platelet count increased to over 150,000/μL, and creatinine decreased to 1.5 mg/dL, which made it possible to discharge the patient with close outpatient monitoring.

Despite the recommendation of one‐month visits for follow‐up and prophylactic plasma therapy, the patient did not return after discharge and was hospitalized again 4 months later due to the recurrence of symptoms (pallor, fatigue, edema, and cola‐colored urine). Laboratory tests showed:

- Hemoglobin: 6.5 g/dL
- Platelets: 60,000/μL
- Creatinine: 5 mg/dL

These findings were consistent with an acute aHUS relapse. Emergent hemodialysis and plasmapheresis were reinitiated; however, the patient demonstrated an inadequate response to plasmapheresis alone. Based on the confirmed CD46 mutation and relapsing course, the decision was made to initiate eculizumab, a terminal complement inhibitor, for targeted therapy.

Following eculizumab treatment, the patient's need for dialysis was resolved, and his blood parameters improved, including the low platelet count. Finally, the patient was discharged in good general condition.

## 5 Discussion

This case report illustrates the potential for COVID‐19 infection to trigger episodes of atypical hemolytic uremic syndrome, particularly in patients with underlying genetic predispositions to complement dysregulation. The homozygous CD46 mutation identified in our patient is particularly noteworthy, as homozygous mutations in this gene are rare in aHUS patients [5].

The CD46 gene encodes membrane cofactor protein (MCP), a key negative regulator of the alternative complement pathway. The identified variant (c.565 T>G, p.Tyr189Asp) is classified as Likely Pathogenic based on the latest ACMG criteria, supported by multiple lines of evidence (PM2, PP3, PP4, PP5). This genetic finding, coupled with the reduced CD46 expression observed on flow cytometry, provides a clear mechanistic link to the patient's clinical presentation.

The connection between COVID‐19 and aHUS has been documented in a few cases. Ville et al. reported a case of aHUS recurrence in a 28‐year‐old female with a heterozygous variant in MCP following COVID‐19 infection [3]. Similarly, Smarz‐Widelska et al. described two related patients with heterozygous MCP/CD46 mutations who experienced aHUS relapse after COVID‐19 infection [4]. Our case adds to this growing body of evidence, suggesting that SARS‐CoV‐2 may act as a trigger for complement dysregulation in genetically susceptible individuals.

The initial management of our patient with plasma therapy, despite the known CD46 deficiency, was based on the urgent need for intervention and the potential benefits of replacing functional complement regulators. However, the relapse and inadequate response to plasmapheresis alone necessitated the use of eculizumab. This decision aligns with the findings in the study, which show that some patients with MCP mutations may require eculizumab treatment, particularly in cases of relapse. The article reports that 4 out of 20 patients received long‐term eculizumab treatment, with 2 patients starting it after multiple relapses. While the study suggests that short‐term or on‐demand eculizumab therapy might be sufficient for many patients with MCP mutations, it acknowledges that individual cases may necessitate its use, especially when faced with recurrent disease episodes [6].

Eculizumab, a humanized monoclonal antibody targeting C5, has transformed the treatment landscape for aHUS. Early initiation of eculizumab therapy provides the best chance for restoring renal function, preventing disease progression, and avoiding critical complications of TMA [7].

In our case, the use of eculizumab led to a significant improvement in the patient's condition, highlighting its efficacy in managing CD46‐associated aHUS.

It's worth noting that whole exome sequencing also revealed a secondary finding of a heterozygous Likely Pathogenic variant in the DNAH9 gene, associated with primary ciliary dyskinesia. While not directly related to the patient's aHUS presentation, this finding underscores the potential for additional genetic insights from comprehensive genomic testing.

## 6 Conclusion

This case report highlights the importance of considering aHUS in pediatric patients presenting with TMA features following COVID‐19 infection, especially in those with a family history or previous episodes of aHUS. Prompt recognition of clinical features, comprehensive genetic evaluation, and initiation of appropriate complement‐targeted therapies are crucial steps in managing aHUS and improving patient outcomes.

The identification of a homozygous CD46 mutation in our patient not only explains the severe clinical presentation but also guides long‐term management strategies. This case adds to the growing evidence of COVID‐19 as a potential trigger for aHUS in genetically predisposed individuals and emphasizes the need for vigilance in monitoring and treating these patients.

Future research should focus on elucidating the precise mechanisms by which SARS‐CoV‐2 interacts with the complement system, particularly in individuals with genetic complement dysregulation. This understanding could lead to more targeted prevention and treatment strategies for COVID‐19‐associated aHUS and other complement‐mediated disorders.

## Author Contributions

**Parsa Lorestani:** methodology, writing – original draft, writing – review and editing. **Parisa Maleki Dana:** writing – original draft. **Mohamad Reza Tohidi:** supervision, writing – original draft.

## Disclosure

This article has been posted to a preprint server.

During the whole process, the patient and his parents were informed about treatment options, risks, and the possibility of relapse. Written informed consent was obtained from the patient's legal guardian for the publication of this case report. A copy of the written consent is available for review by the Editor‐in‐Chief of this journal upon request.

## Conflicts of Interest

The authors declare no conflicts of interest.

## Data Availability

The authors have nothing to report.
